# Combined endoscopic and open frontolateral laryngectomy for glottic tumors involving the anterior commissure

**DOI:** 10.1016/j.bjorl.2025.101742

**Published:** 2025-11-28

**Authors:** Leonardo Haddad, Fabio Pupo Ceccon, Leticia Angélica da Silva Souza, Beatrice Haase Ceccon, João Vitor Pincelli, Mateus Morais Aires

**Affiliations:** a Universidade Federal de São Paulo (UNIFESP), Departamento de Otorrinolaringologia, Cirurgia de Cabeça e Pescoço, São Paulo, SP, Brazil; b Hospital Israelita Albert Einstein, São Paulo, SP, Brazil; c Faculdade de Medicina do ABC, São Paulo, SP, Brazil; d Universidade de Pernambuco (UPE), Faculdade de Ciências Médicas, Recife, PE, Brazil; e Universidade Federal de Pernambuco (UFPE), Hospital das Clínicas, Recife, PE, Brazil

**Keywords:** Laryngeal neoplasms, Laryngectomy, Partial, Surgical procedures, Operative, Voice disorders

## Abstract

**Objective:**

The combined endoscopic and open frontolateral laryngectomy technique has been proposed to balance oncologic safety with functional preservation in the management of glottic tumors involving the Anterior Commissure (AC). This study aims to describe this technique in detail, its indications, and to present our institution’s case series.

**Methods:**

A retrospective review was conducted of all patients undergoing surgery for glottic lesions suspected of malignancy between 2017 and 2024 at a single institution. Patients treated with the combined endoscopic and open frontolateral laryngectomy approach were included. Surgical technique, indications, contraindications, and perioperative management were documented. Outcomes assessed included oncologic control, tracheostomy status, and laryngeal function.

**Results:**

Among 138 patients treated surgically for suspected malignant glottic lesions, four patients underwent the combined approach. Tumor stages included T1b (n = 2), T2 (n = 1), and T3 (n = 1). All patients were male, with a mean age of 71.5-years. Local disease control was achieved in three patients (75%), while one T3 patient experienced recurrence requiring salvage radiotherapy, achieving subsequent disease control. All patients retained laryngeal function, with no need for permanent tracheostomy or reports of dysphagia. Postoperative dysphonia was characterized by roughness, breathiness, and high-pitched but functional voices.

**Conclusion:**

The combined approach offers a feasible alternative for managing AC-involving glottic tumors, providing oncologic safety while preserving function. It enables precise tumor resection with limited sacrifice of normal tissue and the potential for phonation-preserving reconstruction. Given the small sample size, further studies are needed to validate its efficacy and long-term benefits.

**Level of evidence:**

4.

## Highlights

• Combined approach enables precise resection of AC-involving glottic tumors.
• Technique ensures oncologic safety with minimal healthy tissue removal.
• Vocal fold reconstruction may enhance phonatory outcomes.
• No cases of permanent tracheostomy or postoperative dysphagia.
• Careful patient selection is key to optimizing outcomes.

## Introduction

Twenty to twenty-five percent of patients with tumors in the membranous portion of the vocal folds present with Anterior Commissure (AC) involvement.^1^ Glottic tumors that reach the AC have a worse prognosis than those located in the middle third of the vocal fold, as only 2–3 mm separate the vocal ligament from the thyroid cartilage.^2^, ^3^, ^4^ Additionally, because the AC attaches directly to the cartilage, it provides a linear pathway through which malignant cells can invade the thyroid cartilage at a point where there is no internal perichondrium, making it a vulnerable site for tumor progression.^5^^,^^6^

The primary objective of cancer surgery is to achieve adequate disease control and cure. Conservative laryngeal surgery is performed for patients with early-stage laryngeal cancer using approaches such as Transoral Laser Microsurgery (TLM) and open partial laryngectomy. These techniques should only be performed when the surgeon can confidently achieve tumor-free margins.^7^

TLM is a minimally invasive endoscopic approach that combines suspension laryngoscopy with an operating microscope, a tissue-cutting laser, and microsurgical instruments to resect a primary tumor. It is the preferred surgical technique for early-stage tumors and provides an effective alternative to open surgery, offering a similar cure rate and greater preservation of normal tissue, depending on the location and extent of the resection. There are good oncological and phonatory outcomes in T1a, T1b, and T2 tumors.^8^ However, even for early-stages tumors, in cases of vertical involvement of the AC or thyroid cartilage erosion, TLM may not be sufficient, with lower survival rate and higher disease recurrence compared to open partial laryngectomy.^5^^,^^9^, ^10^, ^11^

Open partial laryngectomy is a conservative laryngeal surgery encompassing a broad array of open surgical techniques. Comparing with total laryngectomy, it may be applicable as a strategy to preserve laryngeal function and prevent permanent tracheostomy with its associated negative consequences.^7^ Open frontolateral laryngectomy allows for wide resection of tumors involving the AC, including the vocal muscle, vocal ligament, paraglottic space adjacent to the tumor, inner perichondrium of the thyroid cartilage, arytenoid cartilage, and the anterior portion of the thyroid cartilage.

TLM has largely replaced vertical partial laryngectomies over the past 30-years.^12^, ^13^, ^14^ However, open frontolateral laryngectomy remains a viable option for a limited subset of patients with T1b, T2, and selected T3 tumors, particularly for AC lesions, either due to incomplete tumor exposure during endoscopic surgery or to ensure adequate surgical margins in a single procedure.^15^, ^16^, ^17^ While this approach ensures oncological treatment, an open partial laryngectomy can sometimes be excessively aggressive for small tumors affecting the larynx.

In a combined endoscopic and open frontolateral laryngectomy, the margins are delineated endoscopically, and the anterior portion of the thyroid cartilage is resected externally, along with the tumor tissue adhered to the thyroid cartilage.^18^ This hybrid technique allows for minimal sacrifice of normal tissue, enabling the surgeon to respect the entire specimen under direct visualization. The thyroid cartilage's structural integrity is preserved, and the tumor margins are adequate but not excessive, since endoscopic demarcation can successfully achieve local control with margins of 2 mm,^19^^,^^20^ whereas for open partial laryngectomy, margins of 4–5 mm are recommended.^17^^,^^21^, ^22^, ^23^ Additionally, muscle or mucosal flaps can be utilized to optimize postoperative vocal quality.^24^, ^25^, ^26^

Our objective is to describe this technique in detail, its indications, and to present our institution’s case series.

## Methods

The medical records of all consecutive patients with glottic lesions suspected of malignancy treated in our department between 2017 and 2024 were reviewed. Patients who underwent combined TLM and frontolateral laryngectomy were included. Patients with incomplete medical records were excluded. All surgeries were performed jointly by two authors of this study (LH and FPC) at the same private tertiary community hospital. Ethical approval was obtained from our institution's ethics committee, and informed consent forms were signed by all participants. This study was conducted in accordance with the 2002 World Medical Association Declaration of Helsinki.

### Indications

**Fig. 1:**
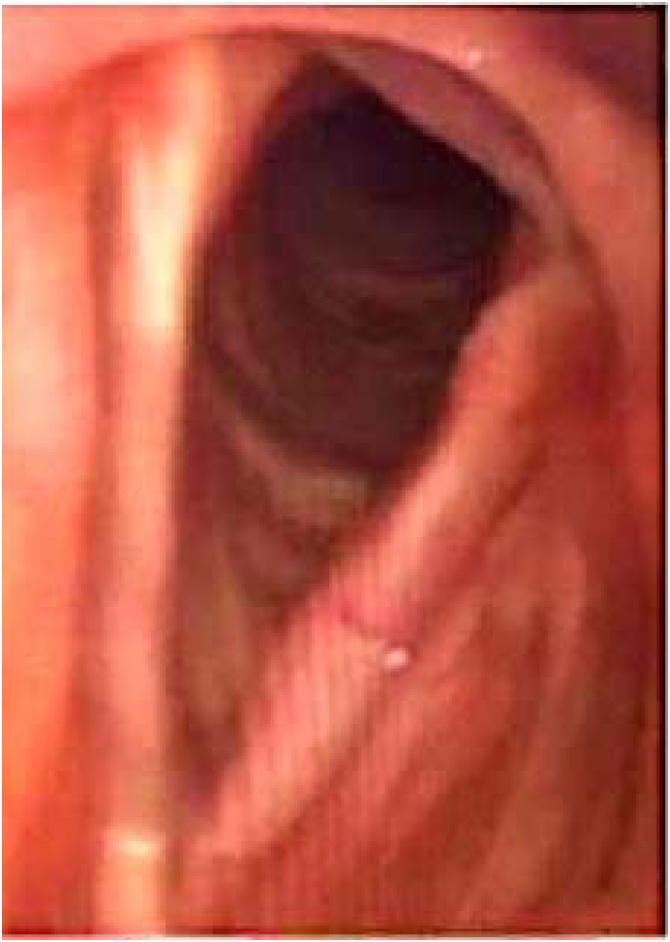
Preoperative laryngoscopy, T1b with larger component on the left.

The indications for the combined approach are the same as those for open frontolateral laryngectomy for T1b, T2, and selected T3 tumors (without evident posterior extension): insufficient anterior margin for endoscopic resection, vertical invasion of the AC, and poor endoscopic exposure of the AC. Contraindications encompass extensive extension into the infraglottic region, thyroid cartilage invasion, and major involvement of the posterior paraglottic space beyond the posterior third of the affected vocal fold, as these conditions typically require more extensive techniques of open laryngectomies. Preoperative assessment includes laryngostroboscopy (Fig. 1), contrast enhanced CT scan with multi-slice imaging, and gadolinium-enhanced MRI of the neck. Thyroid cartilage and paraglottic space invasion are carefully evaluated. Patients must be N0 and M0.

### Technique

Surgery should include removal of the primary tumor along with adjacent and deeper areas susceptible to involvement by the neoplasm, including the adjacent portion of the thyroid cartilage and paraglottic space tissues as a deep margin.

**Fig. 2:**
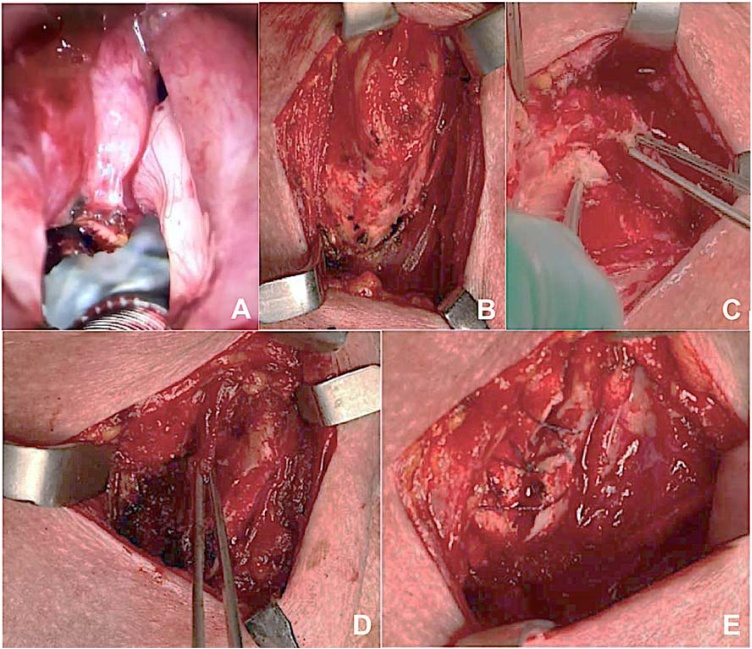
Surgical technique. (A) Left posterior and right anterior cordotomy with CO_2_ laser. (B) The neck is opened and the area where the thyroid cartilage will be removed is marked. (C) Sectioning the thyroid cartilage with a reciprocating saw. (D) Opening of the larynx and rotation of a bipedicle flap of sternohyoid muscle covering the raw area of the tumor resection. (E) Closure of the larynx with polyglactin 3‒0.

The procedure begins with microlaryngoscopy under general anesthesia. A detailed videoendoscopic evaluation is performed using microscopy and 4 mm urology optics from 0 ° to 30 °, assessing tumor boundaries, including the infraglottic and supraglottic areas, hypopharynx, and base of the tongue. Resection of the ipsilateral vestibular fold may be necessary for better exposure. Bilateral posterior cordotomy using a CO_2_ laser is performed to establish the posterior surgical margins, ensuring a 2 mm margin. The limits of the incision are the paraglottic space (deep) and the inner perichondrium of the thyroid cartilage (lateral) (Fig. 2A). Dissection proceeds upward while keeping the specimen attached to the thyroid cartilage in the AC. At this point, the external approach follows with a transverse cervicotomy and exposure of the thyroid cartilage (Fig. 2B). With a reciprocating saw or piezoelectric saw, a bilateral vertical section of the thyroid cartilage is performed, cutting its anterior portion at least 5 mm on each side from the midline of the cartilage (Fig. 2D). The larynx is entered from the least affected side, and the AC that remains attached to the previously endoscopically sectioned piece is resected.

Under direct vision and with the larynx opened, the tumor is removed with the inner perichondrium of the thyroid cartilage. The surgical margins taken from the posterior, upper, and lower regions of the surgical field ‒ not from the resected specimen – are submitted to trans-operative frozen sections examinations. After confirming neoplasm-free margins, reconstruction for phonation is performed using a bipedicle flap of the sternohyoid muscle (Fig. 2D) or a neighborhood flap with the mucosa of the vestibular fold (when preserved), which reduces the raw area of the resection and air leakage during phonation in the postoperative period. The thyroid cartilage is closed with 3‒0 polyglactin sutures (Fig. 2E). Tracheostomy placement is optional but was used in all the patients in this series. All the patients were decannulated before hospital discharge or up to the second week after surgery. Laminar drains were used to prevent subcutaneous emphysema. This finding usually resolves with compressive dressings and a 5-day voice rest.

## Results

Among 138 patients with glottic lesions suspected of malignancy who underwent surgery during the study period, the combined frontolateral laryngectomy was performed in four cases (Table 1). The glottic tumor stages were T1b (n = 2), T2 (n = 1), and T3 (n = 1). All patients were male, the mean age was 71.5 ± 8.9 years (range, 60–81 years). Three patients (75%) had a history of smoking. The follow-up period ranged from 2.3 to 7 years, with local control achieved in 75% of cases. The T3 patient had been clinically staged as T2, but postoperative pathological analysis revealed thyroid cartilage invasion that had not been detected on preoperative imaging. The patient underwent salvage radiotherapy and achieved disease control at the six-month follow-up. Larynx preservation and overall survival rates were 100%.

**Table 1:** Patient characteristics and larynx preservation outcomes.

Patient	Age	T stage	Cartilage invasion	Recurrence	Radiotherapy	Preserved larynx
1	70	T1b	No	No	No	Yes
2	75	T3	Yes	Yes	Yes	Yes
3	60	T1b	No	No	No	Yes
4	81	T2	No	No	No	Yes

**Fig. 3:**
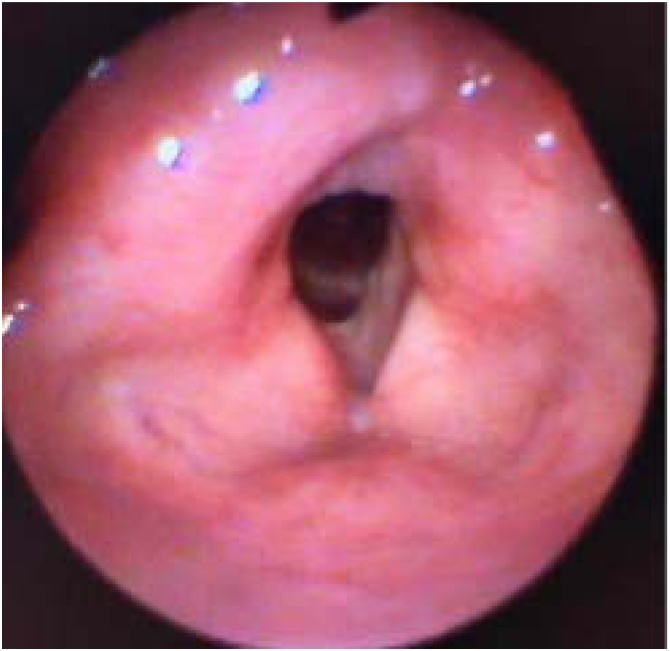
Post-operative findings at 3-year follow-up, demonstrating sustained structural integrity and functional preservation of the larynx.

All patients exhibited postoperative dysphonia characterized by roughness, breathiness, and a high-pitched yet functional voice. None required a permanent tracheostomy, and no cases of dysphagia were reported, demonstrating functional outcomes comparable to those of other partial and endoscopic surgeries for tumors involving the AC. The formation of anterior synechiae is frequently observed in cases requiring extensive manipulation of the AC and the anterior portion of both vocal folds during the same surgical procedure (Fig. 3). This issue is intrinsically related to the tumor, and scarring is an unavoidable consequence of adequate oncologic resection in this anatomically critical region for normal voice production.

## Discussion

Various techniques have been employed to excise glottic carcinomas involving the AC, yet the optimal approach remains a matter of debate. Recently, there has been a growing inclination toward TLM, even for more advanced tumors. However, a significant limiting factor for the endoscopic approach is uncertainty regarding invasion of the thyroid cartilage or the paraglottic space, especially in cases involving the AC with vertical invasion. Additionally, endoscopic surgery can be particularly challenging in AC-involved tumors due to difficult exposure.^27^ Therefore, open partial procedures continue to play an important role in treatment. Partial laryngeal surgery aims to fulfill two essential goals: preservation of the physiological functions of the larynx and complete tumor removal while maintaining oncologic outcomes comparable to those of non-conservative treatments. In Brazil, vertical partial laryngectomies are still widely practiced, with open frontolateral laryngectomy being commonly performed. Local control and survival outcomes have been considered satisfactory.^28^, ^29^, ^30^, ^31^

In an effort to enhance the precision of open techniques ‒ particularly in preserving uninvolved tissues ‒ Shapshay et al.^32^ described, in 1994, a combined endoscopic and open frontolateral laryngectomy in six dogs, termed the “window partial laryngectomy”. Glottic reconstruction was performed using a sternohyoid muscle flap. In 1999, Conticello et al.^33^ reported a series using the combined technique in 28 patients with T1a–T2 tumors, with a minimum follow-up of three years. In 2000, Rebeiz^34^ performed this technique in four patients with T1 glottic carcinoma involving the AC, as a salvage procedure following failed radiotherapy, with a minimum follow-up of 18-months. In 2016, Day et al.^35^ introduced the term “hybrid” approach.

The combined technique offers several advantages. Compared to the purely endoscopic approach, for the indications described, it may provide better local tumor control and potentially superior functional outcomes, due to the possibility of vocal fold reconstruction. Compared to the purely open technique, it allows for greater preservation of healthy tissue ‒ as the required surgical margins are smaller (2 mm vs. 4–5 mm) ‒ and, depending on institutional protocols, may obviate the need for tracheostomy or facilitate early decannulation.

We began performing the combined technique in 2017. In our series, three out of four patients achieved local disease control. Conticello et al.^33^ reported disease-free survival in 27 of 28 patients (96.4%), and Rebeiz^34^ reported 100% disease-free survival in their four cases. Notably, neither study included patients with T3 disease. We included one patient initially classified as T2, who was subsequently found to have T3 disease following histopathological examination of the thyroid cartilage. This patient experienced disease recurrence and required salvage radiotherapy.

Regarding functional outcomes, the combined approach offers the advantage of vocal fold reconstruction, which is not feasible with endoscopic techniques.^36^ Although we did not objectively assess voice quality, all patients retained a functional and communicative voice. Conticello et al.^33^ observed hoarseness in 30% of cases and breathiness in 70%, while Rebeiz^34^ reported good voice quality in all patients. In our series, all patients were successfully decannulated in the early postoperative period. In the study by Conticello et al.,^33^ one patient required a tracheostomy due to chronic respiratory insufficiency, which was closed after six months. In Rebeiz’s series,^34^ no tracheostomies were required. No cases of dysphagia were observed in our cohort, echoing previous findings.

We believe that recent technological advancements have increased the safety of the combined technique, reserving open partial vertical laryngectomies for the most advanced or recurrent cases. These advancements include high-resolution imaging with CT and MRI to assess disease extent, improved intraoperative visualization using video endoscopes with varied angles and lengths, and more refined laser equipment with micro-focus and low-energy settings.

The simplicity of this combined approach ‒ combining the precision and visualization of endoscopy with the completeness of an external resection ‒ aligns with modern surgical goals of minimizing invasiveness and reducing costs. The fact that only four patients have undergone this procedure over a seven-year period highlights the importance of strict and careful patient selection as a key factor for success.

Our experience with the combined approach remains limited to four cases involving T1b, T2, and T3 glottic tumors. Nevertheless, the senior authors of this study have extensive experience with laryngeal surgeries, which lends weight to this report. Even so, the limited sample size is an inherent constraint, precluding a comprehensive assessment of the risks, complications, and oncologic safety of the technique.

## Conclusion

For T1a, T1b, T2, and select T3 glottic tumors involving the AC, the combined endoscopic and open frontolateral laryngectomy represents a viable approach. This technique allows for precise resection margins without compromising oncologic outcomes and may enhance vocal quality by enabling surgical phonation reconstruction. Given the small sample size, further studies are needed to validate its efficacy and long-term benefits. As with other treatments for laryngeal cancer, including partial laryngectomies and wide-field endoscopic resections, patients should undergo regular follow-up to ensure adequate local disease control.

## ORCID ID

Leonardo Haddad: 0000-0003-3392-6259

João Vitor Pincelli: 0009-0009-4687-3150

## Funding

The authors have no funding or financial relationships to disclose.

## Conflict of interest

The authors declare no conflicts of interest.
